# An Efficient Framework for Personalizing EMG-Driven Musculoskeletal Models Based on Reinforcement Learning

**DOI:** 10.1109/TNSRE.2024.3483150

**Published:** 2024-12-03

**Authors:** Joseph Berman, I-Chieh Lee, Jie Yin, He Huang

**Affiliations:** Department of Electrical and Computer Engineering, North Carolina State University, Raleigh, NC 27695 USA; UNC/NC State Joint Department of Biomedical Engineering, North Carolina State University, Raleigh, NC 27695 USA; Unviersity of North Carolina, Chapel Hill, NC 27599 USA; Department of Mechanical and Aerospace Engineering, North Carolina State University, Raleigh, NC 27695 USA; UNC/NC State Joint Department of Biomedical Engineering, North Carolina State University, Raleigh, NC 27695 USA; Unviersity of North Carolina, Chapel Hill, NC 27599 USA

**Keywords:** Reinforcement learning, musculoskeletal modeling, EMG-based neural-machine interface, prostheses

## Abstract

This study aimed to develop a novel framework to quickly personalize electromyography (EMG)-driven musculoskeletal models (MMs) as efferent neural interfaces for upper limb prostheses. Our framework adopts a generic upper-limb MM as a baseline and uses an artificial neural network-based policy to fine-tune the model parameters for MM personalization. The policy was trained by reinforcement learning (RL) to heuristically adjust the MM parameters to maximize the accuracy of estimated hand and wrist motions from EMG inputs. Our present framework was compared to the baseline MM and a widely used MM parameter optimization method: simulated annealing (SA). An offline evaluation was performed to first quantify the time required for MM personalization and the kinematics estimation accuracy of personalized MMs based on data collected from non-disabled subjects. Then, in an online evaluation, additional human subjects, including an individual with a transradial amputation, performed a virtual hand posture matching task using generic and personalized MMs. Results showed that compared to the baseline generic MM, personalized MMs estimated joint motion with lower error in both offline (*p* < 0.05) and online tests (*p* = 0.014), demonstrating the benefit of MM personalization. The RL-based framework performed model optimization in under one second on average in cases that took SA over 13 minutes and yielded comparable kinematics estimations both offline and online. Hence, our present personalization framework can be a practical solution for the daily use of EMG-driven MMs in prostheses or other assistive devices.

## I. Introduction

Musculoskeletal models (MMs) have been useful tools in the field of biomechanics. Historically, these models have been proposed for guiding clinical decision making [1], [2], predicting treatment outcomes [3], and simulating surgical techniques [4]. In several recent studies, electromyography (EMG)-driven MMs have been explored for the purpose of real-time prosthetic device control [5], [6], [7], [8], [9], [10]. MMs have several advantages over EMG-based prosthesis control schemes in current commercial devices. For instance, proportional EMG control or EMG pattern recognition are two popular methods of upper limb prosthesis control that allow users to control the motion of a single degree of freedom at a time [11], [12]. In contrast, MMs allow continuous control of multiple degrees of freedom simultaneously. This allows tasks to be completed more efficiently with coordinated movements of hand and wrist joints. Furthermore, it is likely that they can generalize to new input data significantly better than black-box machine learning approaches like linear regression or artificial neural networks (ANNs) [13], [14]. Yet, while the potential has been shown for MMs to enable reliable and intuitive prosthesis control, there are still no applications of these models for commercial prosthetic devices.

Because individuals, especially those with musculoskeletal impairments, have differences in muscle size, strength, and/or deficits, it is critical to personalize models to guide therapeutic interventions or control assistive devices [15]. However, as these models are often defined with high numbers of musculotendon parameters, building even just a generic MM has been challenging, let alone personalizing the model for individual users. So far, the number of reported methods for personalizing MMs, especially for upper limb models, has been limited. One approach for model personalization is to scale a generic model based on anthropometric measurements. Previous efforts to develop generic MM models, such as those described in [16], [17], [18], and [19], have used published measurements taken from cadavers without musculoskeletal impairments [20], [21]. Then, the anthropometric measurements taken from each user were used to scale the generic model to a personalized one [22], [23], [24], [25]. However, this approach may not be applicable to patients with limb amputations.

In the field of prostheses, EMG-driven MMs have been developed as neural-machine interfaces (NMI) for prosthesis operation. These models typically contain many subject-specific model parameters that cannot be directly measured. Thus, global optimization techniques such as simulated annealing (SA) [26] are commonly used to tailor the model parameters such that the model can optimally estimate the kinematics or kinetics collected from individual users [6], [27], [28], [29], [30], [31], [32], [33], [34]. Nevertheless, techniques like SA can be computationally expensive due to the need for an extensive parameter search and repeated biomechanical simulations, resulting in long optimization times, especially for more complex models with many degrees of freedom and optimizable parameters. For example, SA optimization of 22 parameters of a lumped-parameter upper limb MM developed by our research group was reported to take approximately 20 hours using a Dell Precision T5810 desktop computer with a 3.70 GHz processor and 32 GB RAM [6]. Similarly, another group reported an average of over 20 hours for SA optimization of 102 parameters of an EMG-driven MM of the lower limb using a workstation with an Intel i7 CPU and 8 GB RAM [29]. In addition, occasional parameter recalibrations for these models might be needed to account for the neurophysiological change of residual muscles over time for reasons such as human adaptation and muscle atrophy. Excessive optimization times of a prosthesis controller can thus decrease its usability for daily use. A new solution for the personalization of EMG-driven MMs is needed.

One concept in the field of machine learning for building user-specific data-driven models involves first pretraining a generic model using a large dataset collected from a group of human subjects, followed by quick personalization when individualized data becomes available. For example, the weights and biases of an ANN can be predetermined from scratch from an initial dataset of many users to obtain a model with adequate performance for all users. Then, a select group of these weights and biases may be slightly adjusted using the data of an individual to optimize the model specifically for them. This concept has been used for personalization in multiple applications, such as speech recognition [35], speech enhancement [36], and wearable healthcare technology [37]. The benefit of this approach is that the application always starts with a workable generic model, which can be further tailored toward their users for its optimal operation. Additionally, personalization from a generic model is often data and time efficient compared to learning from a naïve model. This concept and its benefits have inspired us to investigate a new framework for musculoskeletal model personalization, which combines a physiologically informed musculoskeletal model with machine learning.

By leveraging a generic musculoskeletal model, we can use a machine learning approach to quickly achieve model personalization by fine-tuning model parameters based on limited user-specific data. Previously, our group developed a generic EMG-driven upper limb MM using the data collected from a group of non-disabled subjects [38]. We showed that the model allowed multiple new users, including an individual with a transradial amputation, to control a virtual hand in a posture matching task, although this generic model might not necessarily be optimal for each user. Using this generic MM as a starting point, data-driven machine learning methods may further tailor the model parameters toward each individual user. One promising machine learning method is reinforcement learning (RL), which can be used to train a policy to estimate the optimal actions based on the state of the system that most efficiently maximizes its long-term reward [39]. In our application, RL can be used to learn a policy to tune model parameters to maximally approximate the individualized limb dynamics. Compared to search-based optimization algorithms like SA that are often used in the field of biomechanics, RL is based on the Bellman optimality equation [39] and is potentially more robust and time efficient.

Therefore, in this study, we aimed to implement and evaluate our proposed new framework for efficient personalization of EMG-driven MM-based neural-machine interfaces. This framework included our previously developed generic EMG-driven MM of wrist and metacarpophalangeal (MCP) joints [38] and an RL-based policy for MM personalization (RL MM). MMs personalized with our framework were compared with the baseline generic MM and MMs personalized with SA (SA MM) in both offline and online tests. The outcome of this study may inform a novel framework for fast and accurate personalization of EMG-driven MMs as effective NMIs for rehabilitation or assistive device control in the future.

## II. Methods

### A. Subjects

Ten non-disabled (ND) subjects (6 male, 4 female, ages 19–37, right-hand dominant) were first recruited for the collection of training data that were used to pretrain the RL policy. An additional set of six separate ND subjects (5 male, 1 female, ages 21–35, right-hand dominant) and one subject with a transradial amputation (TRA) (male, age 46) were then recruited to complete a virtual hand posture matching task for further evaluation of our optimization method. The TRA subject sustained a left transradial amputation following a traumatic injury. He underwent a targeted muscle reinnervation (TMR) procedure and had used a myoelectric prosthesis with a pattern recognition control scheme for daily activities. The experimental protocol was approved by the North Carolina State University Internal Review Board (protocol number 20882). All subjects provided informed consent prior to their participation.

### B. Training Data Collection

**Fig. 1.**
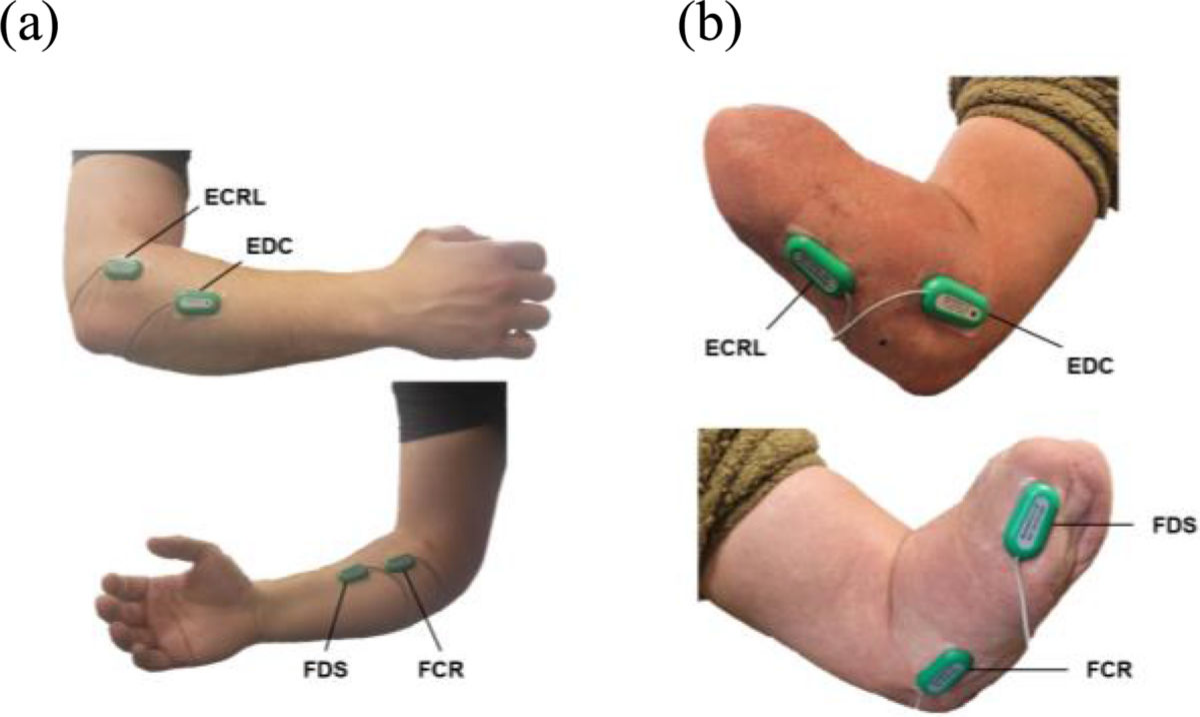
Locations of EMG electrodes for each of the four targeted muscles for an non-disabled subject (a) and the transradial amputee subject (b).

In training data collection sessions, EMG signals and the flexion and extension angles of the wrist joint and metacarpophalangeal (MCP) joint of the middle finger were simultaneously recorded at 1000Hz with a K800 amplifier (Biometrics Ltd., U.K.). Subjects were instructed to move all five fingers of the hand simultaneously to perform hand open and close motions during all data collection sessions. We assumed that the motions of MCP, proximal interphalangeal (PIP), and distal interphalangeal (DIP) joints were coupled. Therefore, only the MCP angle was measured. Four bipolar dry surface electrodes (SX230, Biometrics Ltd., U.K.) were placed over four muscles in the forearm: the extensor carpi radialis longus (ECRL), extensor digitorum communis (EDC), flexor carpi radialis (FCR), and flexor digitorum superficialis (FDS) (Fig. 1). Each muscle was initially identified via palpation and adjusted using visual inspection of the corresponding EMG channel to obtain optimal signal magnitudes. For ND subjects, electric goniometers (SG series and F35, Biometrics Ltd., U.K.) were fixed on the right hand across the MCP joint of the third digit and across the wrist on the posterior side of the forearm. For the TRA subject, the electric goniometers were placed on the intact limb, and they were asked to perform mirrored bilateral movements.

Subjects first performed the maximum voluntary contraction (MVC) of the flexion and extension of each joint. Following this, EMG and joint position data were recorded. During data collection, subjects performed multiple cycles of joint motions, each consisting of an extension and flexion of the wrist followed by extension and flexion of the MCP within a 10s period. For each subject, nine data cycles were recorded and saved for use, totaling 1.5 minutes of data. To regulate the timing of the joint movements, subjects were asked to mirror the motion of a virtual hand displayed on a computer screen during data collection.

### C. Musculoskeletal Model

In this study, a previously developed lumped-parameter musculoskeletal model (MM) was used [6]. The model consists of four Hill-type muscle models representing a wrist extensor and flexor (ECRL and FCR) and a MCP extensor and flexor (EDC and FDS). The Hill-type models each contain a contractile element and parallel elastic element with six total parameters: optimal contractile element length, $l_{opt}$, maximum isometric contractile element force, $F_{0}^{CE}$, wrist moment arm, $ma_{wrist}$, MCP moment arm, $ma_{MCP}$, contractile element length in the neutral posture, $l_{\theta =0}$, and passive elastic element stiffness, $K_{PEE}$. The wrist extensor and flexor muscles do not cross the MCP joint, and thus for those muscles, $ma_{MCP}$ was set to 0. Thus, the model contained a total of 22 optimizable parameters. The values of each model parameter were bound to the ranges listed in Table I.

A previous study with this MM resulted in a set of generic model parameters. A full description of the procedure used to accomplish this can be found in [38]. The generic model parameters were used as the initial values for both optimization methods described in this paper.

Each Hill-type model was driven by a corresponding EMG channel. The recorded EMG signals were first enveloped using the mean absolute value of a sliding window. The sliding window had a length of 200ms and was incremented in 10ms steps, resulting in 100Hz enveloped EMG data. The enveloped data was normalized by the MVC, and for each of the 4 EMG channels, the muscle activation was calculated following the methods used in [13]:

(1) $$u(t)=\alpha e(t-d)-\beta_{1}u(t-1)-\beta_{2}u(t-2)$$

(2) $$a(t)=\frac{e^{Au(t)}-1}{e^{A}-1}$$

where e(t) is the normalized enveloped EMG signal at time t, d is the electromechanical delay set to 40 ms following the methods in [40], α, $\beta_{1}$, $\beta_{2}$ and A are constant values as described in [13], and a(t) is the muscle activation value at time t.

Wrist and MCP joint moments estimated by the Hill-type models were used as inputs to drive a user-generic planar link-segment forward dynamics model to obtain the resulting wrist and MCP joint kinematics. The full details of the implementation of the MM are described in [6].

### D. RL-Based Framework

Using reinforcement learning, we formulated a framework to iteratively update the parameters of the MM. Our goal was to use data collected from the first 10 ND subjects to pretrain a single policy, represented by an ANN, which would then be used to quickly optimize the model parameters for any new subject. For each update step of the optimization procedure, the MM was first used to obtain estimated wrist and MCP joint positions for an entire 10s training data cycle. Features were then extracted from the joint position estimation errors to be used as input to the ANN, which was trained to reduce those estimation errors by outputting a vector of updates for the 22 optimizable model parameters.

#### 1) State Definition:

To define the input state vector at each update step, we considered eight separate timesteps within each 10s cycle of training data consisting of the timesteps of the maximum and minimum values of the measured wrist and MCP joint angles and the midpoints between each of those timesteps. At each of these eight timesteps, the average signed error between the measured and estimated wrist and MCP joint positions, normalized by the approximate joint range of motion (wrist: −75°,75°, MCP: −10°,90°), was calculated in a 500ms window centered at the timestep as follows:

(3) $$e_{i,m}^{w}=\frac{1}{L}\sum_{n=T_{i}-\frac{L}{2}}^{T_{i}+\frac{L}{2}}\left(\theta_{n}^{w}-{\hat{\theta }}_{n}^{w}\right)$$

(4) $$e_{i,m}^{MCP}=\frac{1}{L}\sum_{n=T_{i}-\frac{L}{2}}^{T_{i}+\frac{L}{2}}\left(\theta_{n}^{MCP}-{\hat{\theta }}_{n}^{MCP}\right)$$

where $T_{i}$ is the $i^{\text{th}}$ of the eight previously determined timesteps, $e_{i,m}^{w}$ and $e_{i,m}^{MCP}$ are the average normalized signed error values of the wrist and MCP joint position estimations respectively around $T_{i}$ for update step, m, $\theta_{n}^{w}$ and $\theta_{n}^{MCP}$ are the normalized measured wrist and MCP joint positions respectively at the $n^{\text{th}}$ timestep of the cycle of training data, ${\hat{\theta }}_{n}^{w}$ and ${\hat{\theta }}_{n}^{MCP}$ are the normalized estimated wrist and MCP joint positions respectively at the $n^{\text{th}}$ timestep of the cycle of training data, and L is the number of timesteps equivalent to 500ms (i.e., 50). A 16-element state vector, S, was then formed by concatenating the eight average signed error values for the wrist joint and the eight average signed error values for the MCP joint.

#### 2) Action Definition:

The output action of the ANN at each update step, $A_{m}$, was a vector containing 22 elements, each corresponding to one of the 22 model parameters. The values of $A_{m}$ were bound to the range [−1, 1] by a hyperbolic tangent (tanh) activation function at the output of the ANN. A vector of parameter updates, $\text{Δ}P_{m}$, was obtained by scaling the output of the ANN as follows:

(5) $$\text{Δ}P_{m}=k\left(B_{U}-B_{L}\right)*A_{m}$$

where k is a scaling factor set to 0.05, $B_{U}$ and $B_{L}$ are the upper and lower bounds of the optimizable parameters shown in Table I, and the “*” symbol represents elementwise multiplication. Thus, in each update step, the ANN was able to update each optimizable parameter by up to a maximum magnitude equivalent to 5% of the total range of that parameter.

#### 3) Reward Function:

The reward value in each update step, m, was calculated from the average signed error values as follows:

(6) $$r_{m}=-{\left\|S_{m}\right\|}_{1}=-\sum_{i=1}^{8}\left(\left|e_{i,m}^{w}\right|+\left|e_{i,m}^{MCP}\right|\right)$$

#### 4) Deep Deterministic Policy Gradient:

The deep deterministic policy gradient (DDPG) RL algorithm [41] was used for training. The DDPG algorithm utilizes an agent consisting of an actor ANN with an input state vector, μ(S), to obtain the action vector, A, and a critic ANN with input state and action vectors, Q(S,A), to obtain an estimate of the long-term discounted reward given by the Bellman equation. The actor and critic were designed and trained using the Reinforcement Learning Toolbox in MATLAB 2023a (Mathworks, USA). The actor ANN consisted of an input layer with 16 nodes for the state input, a hidden layer with 100 nodes and rectified linear unit (ReLU) activation function, and an output layer with 22 nodes for the action output and tanh activation function. The critic ANN consisted of an input layer with 16 nodes for the state input and an input layer with 22 nodes for the action input, each followed by a separate hidden layer with 100 nodes. The outputs of these 2 hidden layers were combined with an elementwise addition layer and followed by a 1 node output layer with no activation function. For simplicity, the parameters of the ANNs consisted of only weights with no biases.

At the start of the initial training process, the weights of the actor and critic ANNs, μ and Q, were randomly initialized, and a target actor and target critic ANN, $\mu^{\prime }$ and $Q^{\prime }$, were created with identical structures and weights. Training consisted of a total of 1,000 episodes. At the start of each episode, a new subject from the training data set was selected and the MM was initialized with the generic parameter values. The training procedure cycled through the sets of data collected from each of the initial 10 subjects in a loop such that each subject’s data was used in 100 total episodes. A fixed number of 20 update steps were executed for each episode. Throughout the episode, the training procedure cycled through each provided data cycle of the currently selected subject in a loop, with a new data cycle selected for each new update step. At the start of each update step, the EMG and measured joint position data for the next data cycle of the currently selected subject were loaded. Estimated wrist and MCP joint positions for the data cycle were then obtained from the MM. For update step m, the state vector, $S_{m}$, was calculated using (3) and (4), and the current reward, $r_{m}$, was calculated using (6). The action vector, $A_{m}$, was then obtained from the actor ANN following:

(7) $$A_{m}=\mu \left(S_{m}\right)+N_{m}$$

where $N_{m}$ is a vector of noise sampled from an Ornstein-Uhlenbeck (OU) process [42] to allow for exploration during training. The values of parameter updates were then obtained from (5), and the optimizable parameters, P, were updated as:

(8) $$P_{m+1}=P_{m}+\text{Δ}P_{m}$$

The next cycle of data for the current subject was then loaded and a new state vector, $S_{m+1}$, was calculated from the resulting joint position estimations made by the updated MM. A transition, defined as ($S_{m}$, $A_{m}$, $r_{m}$, $S_{m+1}$), was stored in a first-in-first-out buffer, D. Following this, to update the weights of the ANNs, a minibatch containing N transitions randomly sampled from D was obtained. The weights of the critic were first updated by backpropagation to minimize the loss function:

(9) $$L_{Q}=\frac{1}{N}\sum_{i=1}^{N}{\left(y_{i}-Q\left(S_{i},A_{i}\right)\right)}^{2}$$

where $y_{i}$ is a target value based on the Bellman equation and defined as:

(10) $$y_{i}=r_{i}+\gamma Q^{\prime }\left(S_{i+1},\mu^{\prime }\left(S_{i+1}\right)\right)$$

and γ is the discount factor. Next, the weights of the actor were updated by backpropagation to maximize the long-term discounted reward estimated by the critic by maximizing the policy gradient:

(11) $$\nabla_{\theta^{\mu }}J=\frac{1}{N}\sum_{i=1}^{N}\nabla_{A}Q\left(S_{i},\mu \left(S_{i}\right)\right)\nabla_{\theta^{\mu }}\mu \left(S_{i}\right)$$

where $\theta^{\mu }$ represents the weights of the actor. Finally, the weights of the target actor and target critic were updated to track the weights of the actor and critic using:

(12) $$\theta^{Q^{\prime }}=\tau \theta^{Q}+(1-\tau )\theta^{Q^{\prime }}$$

(13) $$\theta^{\mu^{\prime }}=\tau \theta^{\mu }+(1-\tau )\theta^{\mu^{\prime }}$$

where $\theta^{Q^{\prime }}$, $\theta^{\mu^{\prime }}$, $\theta^{Q}$, and $\theta^{\mu }$ are the weights of the target critic, target actor, critic, and actor respectively, and τ is the target smoothing factor. A full explanation of the DDPG algorithm can be found in [41]. The hyperparameters used in our implementation of DDPG are listed in Table II.

#### 5) Personalization Procedure:

Following the training procedure, the actor ANN, μ, was saved to be used as a policy to optimize the parameters of the MM for new subjects. During the optimization procedure for each new subject, the weights of μ were not updated. Similar to the pretraining procedure, the MM was initialized with the generic parameter values at the beginning of optimization. The optimization procedure again cycled through the total number of provided data cycles in a loop, with a new data cycle selected for each new update step. For each update step, m, an action vector was directly obtained from the actor ANN without added noise as follows:

(14) $$A_{m}=\mu \left(S_{m}\right)$$

The model parameters were then updated by again using (5) and (8). Finally, instead of using a fixed number of 20 update steps, optimization was stopped when convergence in performance was detected. The reward value, $r_{m}$, was calculated at each update step, m, using (3), (4), and (6). The change in reward was tracked as:

(15) $$\text{Δ}r_{m}=r_{m}-r_{m-1}$$

and the average change in reward was calculated using a sliding window with a length of 3 values. The performance was considered to have converged when this average change in reward was nonpositive.

**Fig. 2.**
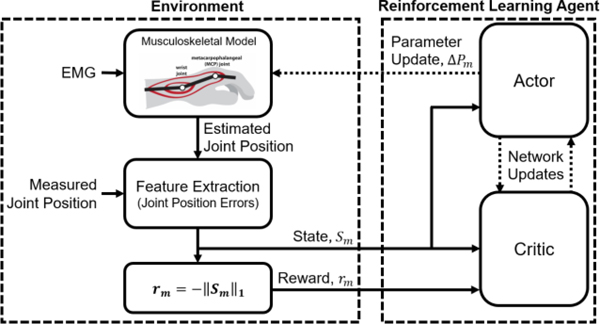
A block diagram of the RL framework. During pretraining, the weights of the actor and critic are updated by the DDPG algorithm. During use, the weights are fixed and only the actor is used to update model parameters until the reward converges.

A block diagram outlining the RL framework is shown in Fig. 2.

### E. Simulated Annealing

To provide a baseline comparison, we considered the simulated annealing (SA) optimization method. At each step of the SA optimization procedure, joint positions were estimated by the MM for the entirety of the provided training data. The measured and estimated joint positions were then each normalized by the approximate joint range of motion, and an objective function was defined as the sum of squares of the normalized estimation errors. At the start of the SA optimization procedure, the MM was initialized with the generic model parameters. Then in each step, the 22 model parameters were normalized to the range [0 1] for input to the SA algorithm. To maximize the speed of the optimization procedure, all code implementing the MM, including the Hill-type muscle models and forward dynamics, were converted to a MEX file. The SA algorithm was implemented using “simulannealbnd” with default settings in the Global Optimization Toolbox with default settings in MATLAB 2023a.

### F. Offline Evaluation

To compare the offline performance of the MMs optimized by the RL policy and SA algorithm, as well as determine the effect of the length of the training data provided to each optimization method, an offline cross-validation evaluation was performed. We considered three different amounts of input training data: one, three, and six data cycles (equivalent to 10, 30, and 60 seconds). For each of the three lengths of training data, a 10-fold cross-validation was conducted.

For each fold, an RL policy was pretrained with data from a different combination of nine subjects, and the resulting policy was used to optimize the MM with the training data from the remaining subject. The length of training data used for each optimization with the remaining subject was equal to the length of training data used to pretrain the given policy. The average optimization time for each method as well as the average time taken to pretrain each RL policy with each length of training data were recorded. SA was then used to optimize a separate MM with the same training data from the remaining subject. Using a separate set of testing data from the remaining subject containing three data cycles, estimated joint positions were obtained from the MMs optimized with each method using each length of training data, as well as the MM with the generic model parameters. The normalized root mean square error (NRMSE) between the measured and estimated joint positions was calculated by normalizing the root mean square error by the approximate joint ranges of motion. The offline NRMSE was averaged across both joints and all subjects for each optimization method and number of data cycles used. Finally, for each optimization method, we considered the average change in model parameters from their initial values across all lengths of training data, measured as the absolute difference of the value of each model parameter before and after optimization, normalized by its respective range.

Both optimization methods were executed on a laptop computer (2.20GHz Intel i7 processor, NVIDIA GeForce RTX 3070 GPU, 16GB RAM).

### G. Virtual Hand Posture Matching Task

To evaluate the real-time task performance of subjects using the optimized MMs, we used a real-time virtual posture matching task similar to what was used in previous studies [14], [38], [43], [44]. Six ND subjects and the TRA subject, whose data were not used to pretrain the RL policy, were recruited for a data collection session involving this task.

At the beginning of each session, the EMG electrodes and electric goniometers were placed, and EMG signals were recorded while subjects performed the MVC for the flexion and extension of each joint following the methods described in Section II-B. Following this, training data were collected from each subject. For the purpose of the real-time task performance evaluation, a single RL policy was pretrained using three data cycles from each of the 10 initial ND subjects and three training data cycles were collected from each additional subject in the online evaluation for use in both optimization methods. After optimization with each method, the resulting set of model parameters was saved for use in the task.

**Fig. 3.**
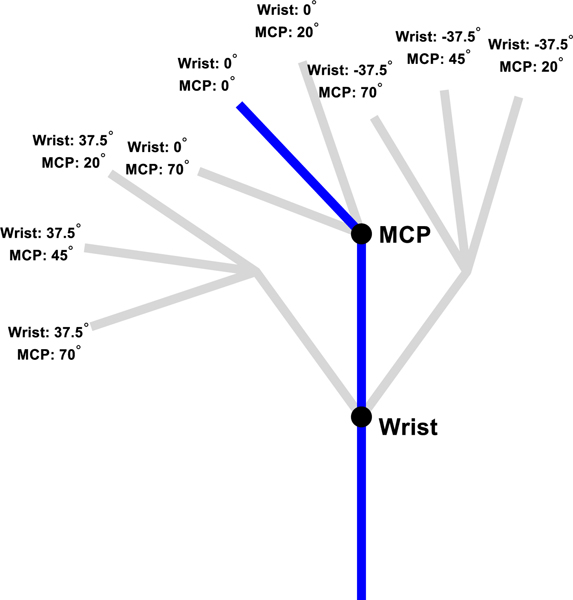
The 8 target postures (gray), representing various combinations of flexion and extension positions of the wrist and MCP joints, and the base posture (blue), used as the starting position for each attempt to match a posture.

For this task, a black-colored virtual 2-DOF planar link-segment hand visualizing the wrist and MCP joint angles was displayed with a 20Hz refresh rate on a computer screen on table in front of subjects. In a single trial of the task, 8 gray-colored target postures representing various combinations of joint flexion and extension positions were given to the subject in a random order. While each target posture was displayed, muscle activations were computed in real time from recorded EMG signals and the resulting joint angles estimated by the MM were used to continuously update the virtual hand. Subjects were given 10 seconds to match the position of the virtual hand to each target posture. A target posture was considered completed when the virtual hand was held within a tolerance of ±5% of the approximate range of motion of each joint (wrist: ±7.5°, MCP: ±5°) for 0.5 consecutive seconds. The color of the virtual hand changed to green when it was within this range. To reset the position of their physical hand before the start of each new target posture matching attempt, subjects matched the virtual hand – updated with joint angles measured by the electric goniometers - to a base posture (wrist: 0°, MCP: 45°). The base posture was considered complete using the same criteria as for the target postures, but the 10 second time limit was not enforced. The positions of the 8 target postures and base posture are visualized in Fig. 3.

Subjects completed a series of trials using each of 3 different MMs: the generic MM, the RL MM, and the SA MM. The order in which the MMs were given was randomized for each subject. For each MM, similar to the methods used in [43], [44], subjects were first asked to perform 5 practice trials to allow them to adapt to the task with the given MM. The number of target postures successfully completed was recorded as a measure of task performance in each trial, and after the first 5 practice trials, a line was fitted to these values. An Ftest was used to determine if the slope of the fitted line was statistically different from 0, indicating that the subject’s task performance was still changing, or statistically similar to 0, indicating that the subject’s task performance had converged. If task performance was determined to be still changing, an additional practice trial was given, and a new line was fitted to the last 5 trials to recheck for convergence in the subject’s task performance. If task performance was determined to be converged, the subject proceeded to complete 5 evaluation trials with the given MM.

While ND subjects attempted to complete each target posture, the joint angles measured by the electric goniometers and the joint angles estimated by the MM were simultaneously recorded and saved. The NRMSE between these measured and estimated joint positions was averaged across both joints and all ND subjects as a measure of the accuracy of the real-time joint angle estimations made by the MM. Task performance of ND subjects was quantified by the averaged completion rate of target postures in each evaluation trial for each MM was used.

For the TRA subject, joint angles were not recorded by the electric goniometers while attempting to complete the target postures to avoid excess mental burden from completing mirrored bilateral movements during the task. Additionally, to account for the difference from ND subjects in task performance, we considered two metrics. The first was the average number of target postures in which the virtual hand was held within a tolerance of ±10% of the approximate range of motion of each joint (wrist: ±15°, MCP: ±10°) for 0.5 consecutive seconds. The second – identical to the criteria used for ND subjects – was the average number of target postures in which the virtual hand was held within ±5% of the approximate range of motion of each joint for 0.5 consecutive seconds.

### H. Statistics

Before running the statistical comparison, the Shapiro-Wilk normality test was performed. If the dataset used for the statistical test was not normally distributed, a non-parametric test was applied. For offline evaluations, to compare the offline NRMSE values, a two-way repeated measures ANOVA was applied (3 types of MM: generic MM, RL MM, and SA MM × 3 training cycles: 1, 3, and 6 data cycles). To compare the absolute changes in parameter values, a three-way repeated measures ANOVA was applied (3 types of MM: generic, RL MM, and SA MM × 3 training cycles: 1, 3, and 6 data cycles × 22 model parameters).

For the online evaluation, to compare the numbers of target postures completed, a one-way repeated measures ANOVA was applied using the three types of MMs. Online NRMSE values were not normally distributed, so a non-parametric Friedman test was used to compare the three types of MMs (generic, RL MM, and SA MM).

For all post-hoc analyses, the Bonferroni correction was applied. Since the online NRMSE values were not normally distributed, Wilcoxon signed-rank tests were conducted for the post-hoc analysis with Bonferroni correction. The significance level for all tests was set at α=0.05.

## III. Results

### A. Offline Evaluation Results

**Fig. 4.**
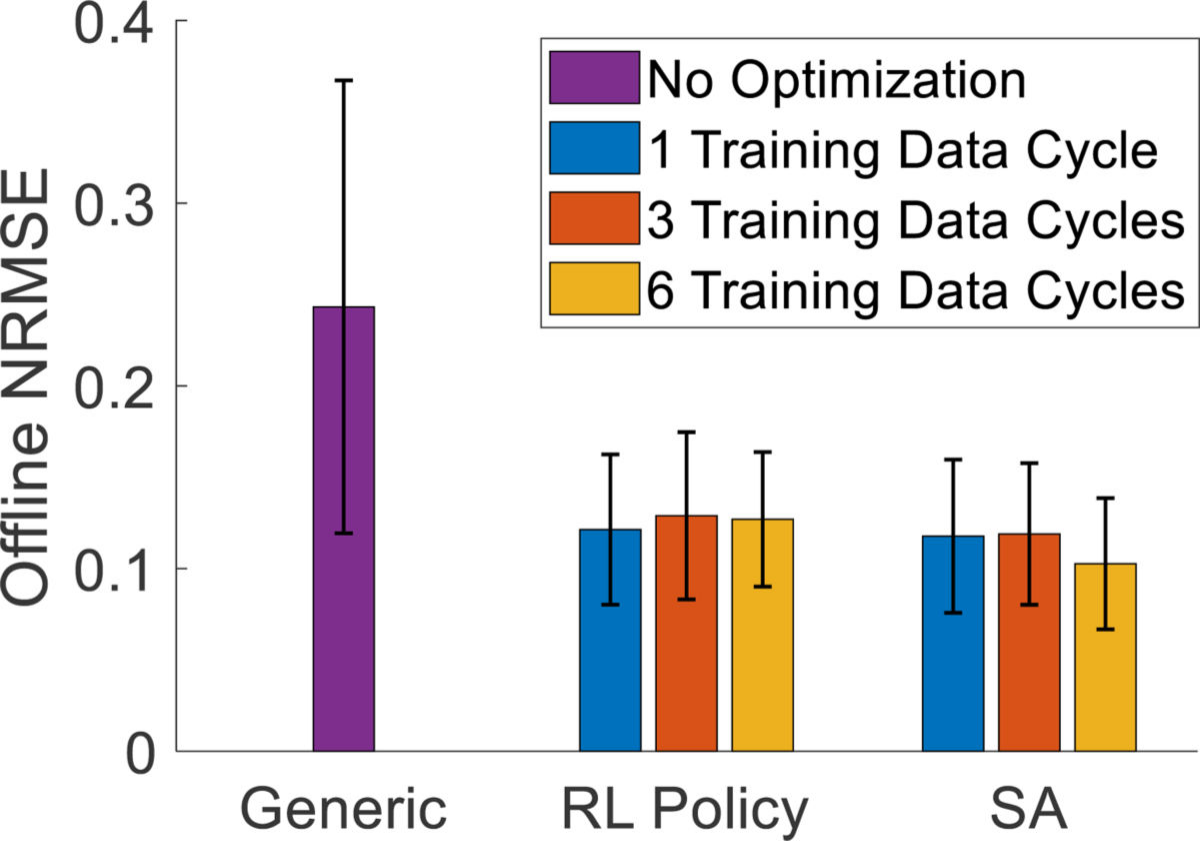
The average NRMSE values achieved by the generic musculoskeletal model (left), the RL policy-optimized musculoskeletal model (middle), and the SA-optimized musculoskeletal model (right). The values for each optimization method represent the average NRMSE achieved after training with 1 data cycle (blue), 2 data cycles (orange), or 3 data cycles (yellow). Error bars represent standard deviation.

For the offline cross-validation evaluation, RL policies were pretrained using one, three, and six data cycles taking 888.5±100.1s, 906.7±99.4s, and 901.9±107.9s respectively. The generic MM achieved an average NRMSE value of 0.24±0.06 when evaluated with the testing data. The MMs optimized by the RL policies using one, three, and six training data cycles achieved average NRMSE values of 0.12±0.03, 0.13±0.02, and 0.13±0.02 respectively. The MMs optimized by SA using one, three, and six training data cycles achieved average NRMSE values of 0.12±0.02, 0.12±0.03, and 0.10±0.02 respectively. There was no interaction effect between the optimization method and data cycles (p = 0.414). The main effect showed that the type of MM reached a significant difference (p < 0.05), but the number of training data cycles did not (p = 0.631). Post-hoc showed that both the RL MM and the SA MM were significantly lower than generic (p < 0.05). However, no significant differences were found between the RL MMs and the SA MMs (p = 0.067). The average NRMSE values are shown in Fig. 4. Additionally, the average optimization time taken, and average number of updates steps executed by the pretrained RL policies and SA using each length of training data are shown in Table III and Table IV respectively.

**Fig. 5.**
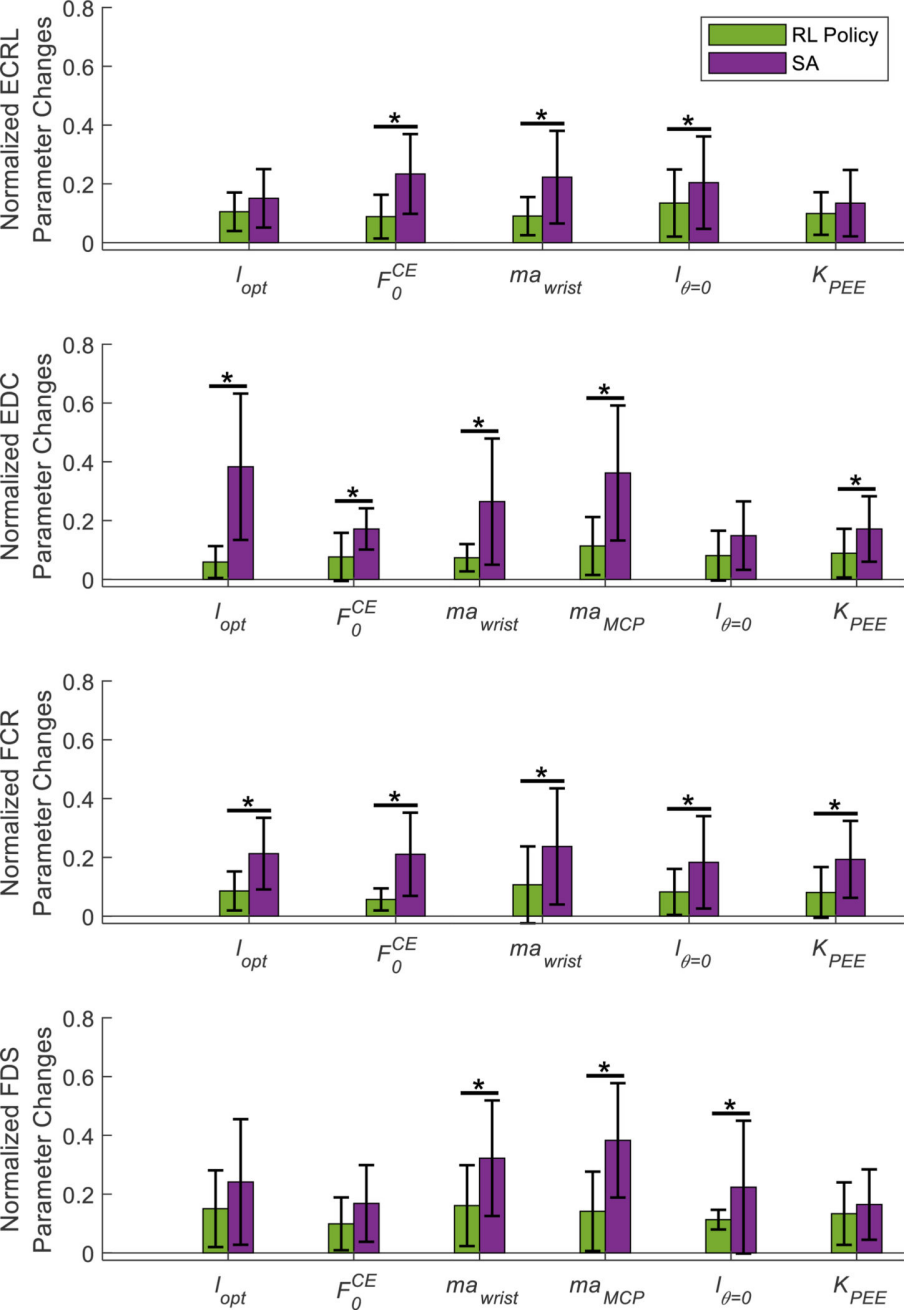
The magnitudes of change in model parameters from the generic musculoskeletal model, normalized by their respective ranges, for each of the four Hill-type models after optimization with an RL Policy (green) and SA (purple) using three training data cycles. Error bars represent standard deviation. Stars indicate statistically significant differences.

The average absolute change in the value of each model parameter from the initial generic model parameter values after optimization by the RL policies and SA across all lengths of training data, normalized by the respective range of each parameter, is shown in Fig. 5. Significance was found for the effects of optimization method (p < 0.01), the number of training cycles (p < 0.01), and the model parameter (p < 0.01) on the absolute change in model parameter value. Post-hoc analysis showed that the RL polices resulted in significantly lower changes in parameter values than SA (p < 0.05). Optimizations performed using six data cycles also resulted in smaller changes in parameter values than using one data cycles (p < 0.05), while there was no significant difference in the changes in parameter values between optimizations using one and three data cycles (p = 0.142). Additionally, there was a significant interaction between the optimization method and model parameter (p < 0.05). The post hoc analysis showed that changes in parameter values made by RL policies were significantly lower for 16 out of the 22 model parameters compared to changes in parameter values made by SA (p < 0.046) as shown in Fig. 5.

**Fig. 6.**
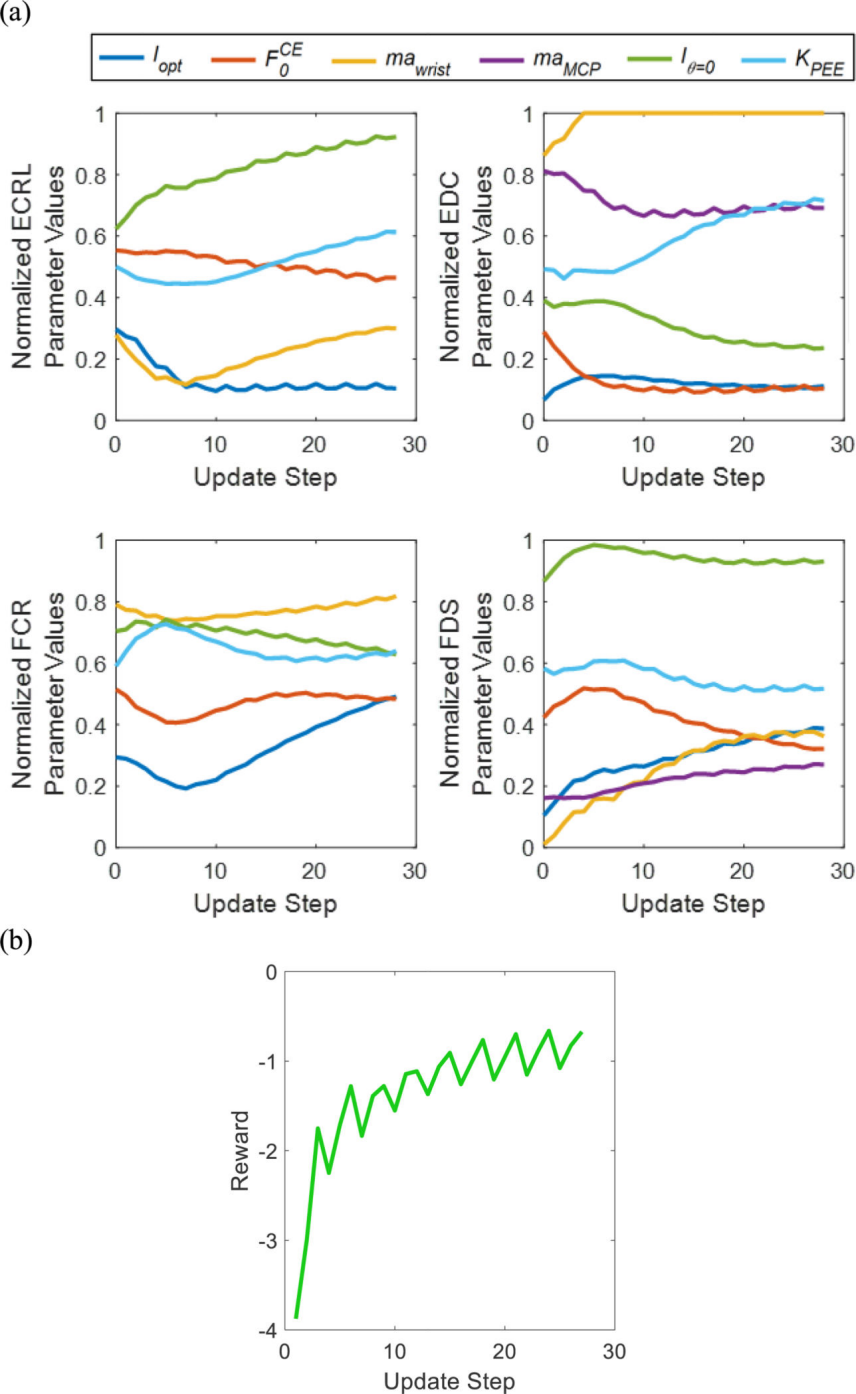
Results from the optimization of model parameters by an RL policy using data collected from an non-disabled subject. (a) The values of model parameters, normalized by their respective ranges, for each of the four Hill-type models at each update step. The musculoskeletal model is initialized with the generic model parameters at step 0. (b) The reward value resulting from the parameter updates applied at each step. The optimization procedure finished when convergence of the reward was detected.

**Fig. 7.**
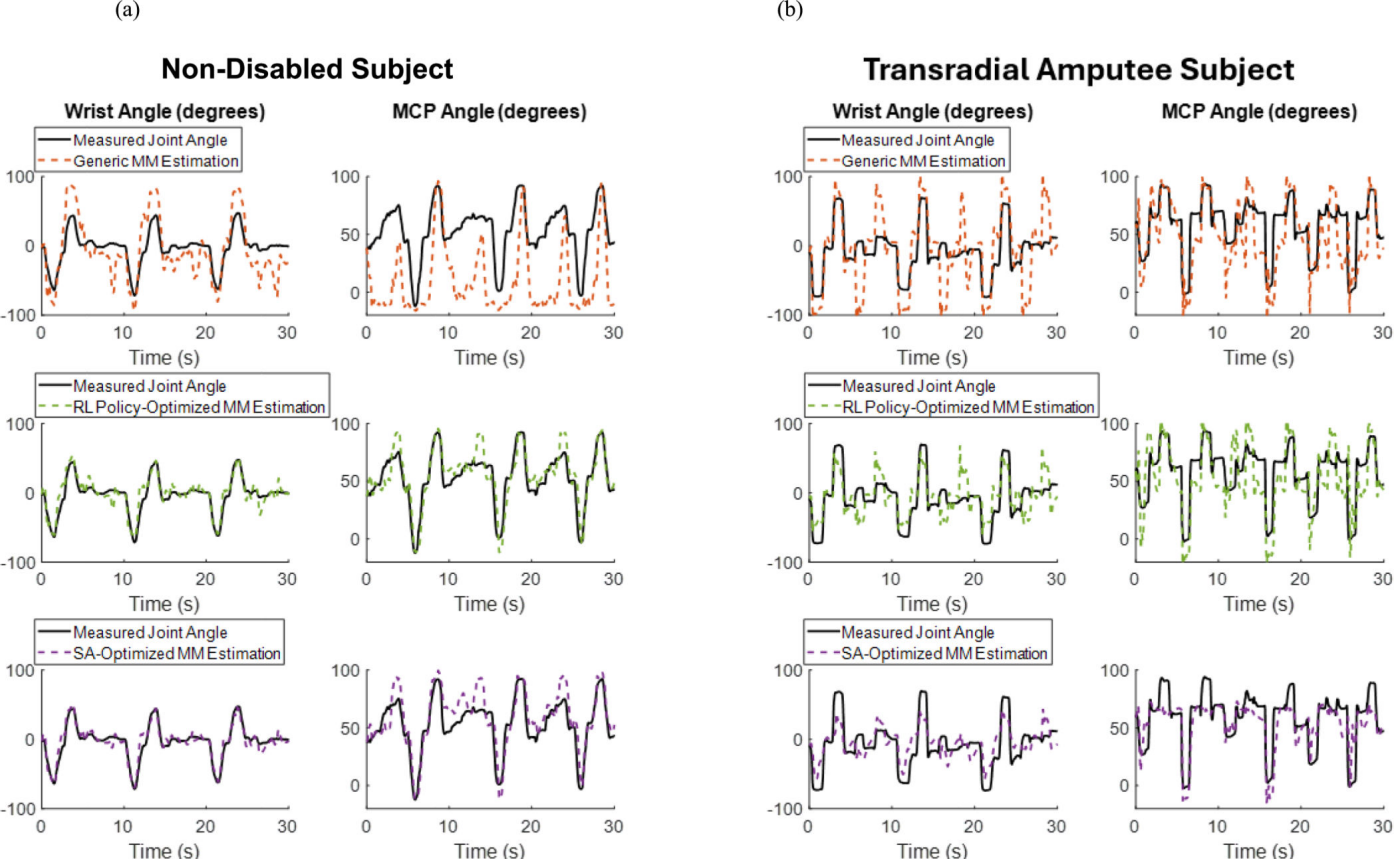
Measured and estimated wrist (left) and MCP (right) joint angles for the testing data collected from an non-disabled subject (a) and transradial amputee subject (b). The estimated joint angles shown were obtained from musculoskeletal models with generic model parameters (top), model parameters optimized by the RL policy with 3 training data cycles (middle), and model parameters optimized by SA with 3 training data cycles (bottom).

Fig. 6 shows an example visualization of the change in model parameters, as well as the resulting reward values, at each step of the optimization procedure with an RL policy using 3 training data cycles collected from an ND subject. The optimization procedure is stopped at the detection of the convergence of the reward (Fig. 6b). The joint position estimations made by MMs with the generic model parameters, model parameters optimized by the RL policy with 3 training data cycles, and model parameters optimized by SA with 3 training data cycles for the testing data collected from the same ND subject and testing data collected from the TRA subject, are shown in Fig. 7.

### B. Virtual Hand Posture Matching Task Results

**Fig. 8.**
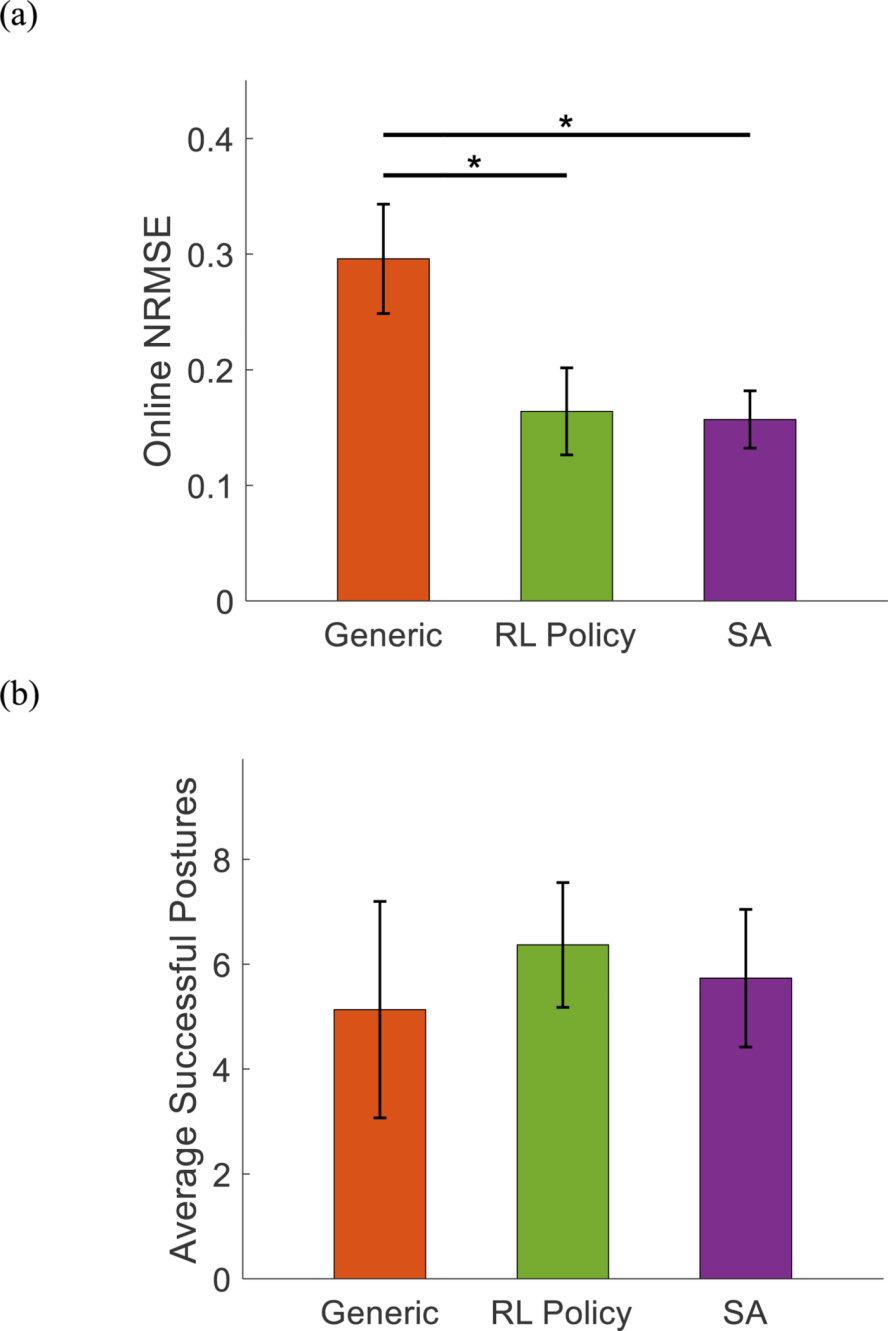
The average virtual posture matching task results for non-disabled subjects using musculoskeletal models with generic model parameters (orange), model parameters optimized by the RL policy (green), and model parameters optimized by SA (purple). (a) The average NRMSE between measured and estimated joint positions while attempting to complete postures with each musculoskeletal model. (b) Task performance quantified as the average number of target postures successfully completed. Error bars represent standard deviation. Stars indicate statistically significant differences.

The average online NRMSE between measured and estimated joint positions from the generic MM, RL policy-optimized MMs, and SA-optimized MMs across eight target postures were 0.30±0.05, 0.16±0.04, and 0.16±0.02 respectively (Fig. 8a). The type of optimization method used had a significant effect on the online NRMSE (p = 0.011). Compared to the generic MM, online NRMSE was significantly lower for both the RL MMs (p = 0.014) and SA MMs (p = 0.014). However, online NRMSE was not significantly different between RL MMs and SA MMs (p = 0.753).

Additionally, ND subjects using the generic MM, RL policy-optimized MM, and SA-optimized MM were able to complete 5.1±2.1, 6.4±1.2, and 5.7±1.3 target postures respectively during the virtual hand posture matching task (Fig. 8b). The type of optimization method used had a significant effect on the number of target postures completed (p < 0.05). However, the post hoc analysis showed that no significant differences were found between the generic MM, RL MM, or SA MM (p >0.069).

Finally, the average task performance achieved by the TRA subject using each MM, quantified as the number of postures considered complete using both the ±10% and ±5% tolerance in the range of motion criteria, is shown in Table V.

## IV. Discussion

In this study, we introduced a novel framework for efficient personalization of EMG-driven MMs used as neural-machine interfaces for prostheses. For this framework a reinforcement learning (RL) algorithm was used to establish a policy to fine-tune parameters of a generic MM using data collected from a group of ND subjects. The learned policy was then evaluated with additional ND subjects and a subject with a transradial amputation (TRA) not included in the training data. Using the generic MM as a baseline for comparisons, we evaluated the benefits of personalization by our RL-based framework and a popular global optimization algorithm: simulated annealing (SA). The generic and personalized MMs were compared in terms of performance in an offline evaluation and online evaluation using a virtual hand posture matching task. Additionally, we considered the differences in required optimization time between our framework and SA to evaluate the practicality of each method for personalizing EMG-driven MMs for prostheses and assistive devices.

Compared to the baseline generic MM, the MMs personalized by either method achieved significantly better kinematics estimation accuracy for the offline evaluation (Fig. 4). For the online virtual hand posture matching task, personalized MMs also yielded significantly lower errors in estimating joint kinematics and a slightly higher task completion rate (Fig. 8). This result has strong implications of the benefits of personalizing MMs used as neural-machine interfaces. Although the generic MM was functional with new users, as we observed in the previous study [38], the personalized MM was shown to more closely match the intended motions of users, which can potentially improve the ability of users to adapt to the NMI and reduce muscle efforts while using the NMI for real-time task performance [43], [44]. Our future work will test more human subjects and real-time tasks to quantify the benefit of personalizing MM-based neural-machine interfaces in terms of users’ physical and cognitive functions.

While personalization of MMs with either our RL-based framework or SA resulted in similar performance for the offline and online tests, our framework yielded much faster MM parameter personalization than the SA algorithm, as shown in Table III. The reason is that the policies were able to form more efficient parameter tuning strategies by pretraining using RL with the data collected from multiple previous subjects. In the RL pretraining procedure, policies learn to make parameter adjustments that maximize the long-term value of reward for many different possible model states. Therefore, the final trained policies may not need to extensively explore the entire parameter space for every new user, but they can instead more efficiently adjust parameter values based on previous experience. By contrast, traditional search-based global optimization algorithms like SA must sufficiently search through the entire parameter space every time an MM is personalized to a new user to ensure that the global optimum is found. It is likely for this reason that although both personalization methods were initialized with the same generic model parameters, the changes made to most parameter values were significantly smaller when using our RL-based framework than when using SA (Fig. 5). This result shows that the RL MMs by fine-tuning the initial generic parameters, while SA may find much different solutions for each user. Because the performance of the personalized MMs was similar regardless of the method used, the lumped-parameter MM used in this study likely had multiple combinations of model parameter values for each subject that could result in optimal performance for our specific offline and online tests. Potentially, the solution can be further limited when including additional constraints, such as tighter parameter ranges, or more complex testing tasks, such as operating prosthetics to perform functional tasks. This can be investigated in our future work. Finally, the ability of the RL-based framework to personalize EMG-driven MMs significantly faster than traditional methods can also greatly improve the adaptability and usability of MMs as NMIs for day-to-day assistive device operation. Users can potentially run a quick MM parameter personalization each time they use their prosthesis for optimal operation.

Personalization of the MM with either optimization method also appeared to slightly improve the TRA subject’s performance in the virtual hand posture matching task (Table V). However, his overall average task performance was lower than that of ND subjects. This may be partly due to the mirror bilateral movements during training data collection, in which his intent for moving the amputated arm was approximated by the intact limb motion. Another potential reason is a significant variation in the lengths and locations of residual muscles, which presented an additional challenge in determining EMG electrode placements to record isolated activity from each targeted muscle. It was observed that during each attempt to match a target posture, the TRA subject was able to consistently move each joint in the direction they desired but had difficulty holding the virtual hand still near the target posture for the required time period. Thus, the number of target postures successfully completed was much higher when considering the higher tolerance for each. However, in our further work with this MM, it may be possible to improve prosthesis control for amputee subjects by utilizing techniques such as filtering the estimated joint motions or preventing movement of the prosthetic device for any sufficiently small estimated joint movement. Additionally, it should be noted that this performance was achieved after only a limited number of practice trials. Daily use may allow subjects to further improve their performance with personalized MMs. Future work with the RL framework should incorporate amputee data in the training datasets for policies to improve robustness to input EMG signal variations and include more individuals with amputations in both offline and online evaluations. Nonetheless, the promising initial results in this present study imply that our framework may in the future be applicable to prosthetic device users with muscle deficits.

There are also several limitations from this study that should be considered. First, because our proposed framework involves fine-tuning existing generic model parameters rather than searching through the entire parameter space, it is not guaranteed to find the true global optimal solution every time. For example, our results showed that many model parameters found by the RL framework were significantly different from SA. In this case, the resulting performance of models personalized by either method was similar, suggesting that multiple globally optimal solutions may exist for each subject. However, future work focusing on more complex models with larger solution spaces should consider the risk that solutions are a local rather than global minima. This risk can be mitigated by ensuring an adequately performing initial generic model, allowing the RL policy to sufficiently explore the parameter search space during pretraining, and carefully designing the convergence detection for personalization. Second, for this proof of concept of our proposed framework, we collected a small pretraining dataset from only 10 ND subjects. While our framework was successful for the evaluations in this study, including more training data from more subjects, especially those with transradial amputations, will likely improve the robustness of the framework to variations in EMG signals. Additionally, it may be possible to explore data augmentation methods to modify the collected EMG and joint kinematics data to artificially expand the dataset used to pretrain policies. Next, the online evaluation conducted for this study was limited. Although the error of joint position estimations made in real time during the virtual hand posture matching task was significantly reduced with model personalization, the number of successfully completed postures was not significantly different. This may be because the chosen task and performance metric were not sufficient to fully highlight the benefits of MM personalization. Many additional metrics, such as cognitive load and user adaptation rate, can be used to quantify the real-time task performance of users of an EMG-based NMI. More online tasks can be explored, and more subjects, including patients with transradial amputations, can be included in the evaluation. Finally, the lumped-parameter MM used in this study is relatively simple, containing only two degrees of freedom and 22 optimizable model parameters. A model with only two DOF does not account for multiple finger movements. Future work may explore the benefits of our framework with more complex MMs that consider the motion of individual fingers separately. Additionally, MMs of the lower limb that require higher numbers of muscle models capable of greater magnitudes of force can be considered. More complex EMG-driven MMs with more degrees of freedom, muscle models, and parameters will likely require a larger amount of data for effective personalization. To accurately describe the state of the model, this work may additionally need to implement extensive feature extraction methods, or if appropriate for the available training data, apply machine learning methods like convolution neural networks.

## V. Conclusion

In this study, we presented a novel framework to quickly personalize EMG-driven musculoskeletal models (MMs) for use as neural-machine interfaces. The framework combines a previously developed generic musculoskeletal model (MM) with a machine learning-based method of fine-tuning model parameters for individual users. To investigate the benefits of this new framework, we compared the initial generic MM with MMs personalized by either our RL-based framework or a traditional optimization technique used for MM personalization: simulated annealing (SA). Our evaluation included an offline analysis of the accuracy of hand and wrist motions estimated by MMs using data collected from non-disabled (ND) subjects, followed by an online virtual hand posture matching task with additional ND subjects and a subject with a transradial amputation. Our results suggest that personalizing a generic MM, either with our RL MM or SA MM, can improve offline and online performance by similar amounts. However, the RL personalized MMs significantly faster than SA. Thus, this proposed framework may be considered as a potential option for efficient personalization of EMG-driven MMs designed for prosthesis control in daily practice. This concept of using a machine learning agent can be implemented with a variety of possible training algorithms and feature extraction methods and applied to a range of different MMs including both upper and lower limb models. In the future, this framework may be also extended to other rehabilitation applications of MMs in which model personalization is essential.

## Figures and Tables

**TABLE I:** Lower and Upper Bounds for Each Model Parameter

Model Parameter	Lower Bound	Upper Bound
Optimal Contractile Element Length, $l_{\text{opt}}(\text{m})$	0.1	0.5
Maximum Isometric Contractile Element Force, $F_{0}^{CE}(\text{N})$	10	1000
Wrist Moment Arm, $ma_{wrist}(\text{m})$	0.001	0.05
MCP Moment Arm, $ma_{MCP}$* (m)	0.001	0.05
Contractile Element Length in the Neural Posture, $l_{\theta =0},\left(\%\;l_{opt}\right)$	75	125
Passive Elastic Element Stiffness, $K_{PEE}\left(\text{N}/{\text{m}}^{2}\right)$	10	200

* $ma_{MCP}=0$ for ECRL and FCR Muscle Models

**TABLE II:** Hyperparameters for the DDPG Algorithm

Hyperparameter	Value
Learning Rate	10^−3^
Discount Factor, γ	0.99
Target Smoothing Factor, τ	10^−3^
L2 Regularization	0
Experience Buffer Size	4,000
Minibatch Size	100
Standard Deviation of OU Process	0.30
Standard Deviation Decay Rate of OU Process	10^−4^
Mean Attraction Constant of OU Process	0.15

**TABLE III:** Average Optimization Times for Each Length of Training Data

	
	Average Optimization Time (s)		

	1 Data Cycle	3 Data Cycles	6 Data Cycles
**RL Policy**	0.34 ± 0.15	0.33 ± 0.16	0.22 ± 0.05
**SA**	64.64 ± 23.56	276.89 ± 113.93	787.84 ± 251.49

**TABLE IV:** Average Number of Update Steps Executed for Each Length of Training Data

	
	Average Number of Update Steps		

	1 Data Cycle	3 Data Cycles	6 Data Cycles
**RL Policy**	12.5 ± 6.6	11.6 ± 6.8	7.3 ± 2.1
**SA**	19,368.6 ± 6,394.7	29,413.6 ± 11,816.9	44,085.2 ± 13,949.4

**TABLE V:** Average Task Performance of the Transradial Amputee Subject

	
	Average Successful Postures		

	Generic	RL Policy	SA
**±10% Tolerance**	3.0 ± 3.9	4.2 ± 4.0	4.2 ± 4.0
**±5% Tolerance**	0.4 ± 1.8	1.2 ± 2.9	0.2 ± 1.3
